# A method of motion estimation of segmental ventricular wall with tracking of ultrasonic echocardiogram

**DOI:** 10.1186/s12880-023-01040-3

**Published:** 2023-07-05

**Authors:** Shanna Liu, Hao Zhang, Chang Li, Weifang Dai, Jinyu Wu, Yuanyuan Wu, Wenwen Su, Bin Xia, Jiayu Zhou, Yuqiang Shen, Xinjian Zhu

**Affiliations:** 1 Department of Information Technology, The Fourth Affiliated Hospital, Zhejiang University School of Medicine, Yiwu, Zhejiang 322000 China; 2 Department of Cardiology, The Fourth Affiliated Hospital, Zhejiang University School of Medicine, Yiwu, Zhejiang 322000 China; 3 Department of Ultrasonography, The Fourth Affiliated Hospital, Zhejiang University School of Medicine, Yiwu, Zhejiang 322000 China; 4 Xinjiang Second Medical College, Karamay, Xinjiang 834000 China

**Keywords:** Motion estimation, Segmental ventricular wall, Optical flow tracing, Ultrasonic echocardiogram

## Abstract

**Background:**

Ultrasonic echocardiography is commonly used for monitoring myocardial dysfunction. However, it has limitations such as poor quality of echocardiography images and subjective judgment of doctors.

**Methods:**

In this paper, a calculation model based on optical flow tracking of echocardiogram is proposed for the quantitative estimation motion of the segmental wall. To improve the accuracy of optical flow estimation, a method based on confidence-optimized multiresolution(COM) optical flow model is proposed to reduce the estimation errors caused by the large amplitude of myocardial motion and the presence of “shadows” and other image quality problems. In addition, motion vector decomposition and dynamic tracking of the ventricular region of interest are used to extract information regarding the myocardial segmental motion. The proposed method was validated using simulation images and 50 clinical cases (25 patients and 25 healthy volunteers) for myocardial motion analysis.

**Results:**

The results demonstrated that the proposed method could track the motion information of myocardial segments well and reduce the estimation errors of optical flow caused due to the use of low-quality echocardiogram images.

**Conclusions:**

The proposed method improves the accuracy of motion estimation for the cardiac ventricular wall.

## Background

Cardiovascular diseases (CVDs) are a major threat to human health and the leading cause of death [1, 2]. Among them, ischemic heart disease is a CVD that accounts for the largest proportion [3]. The main cause is myocardial ischemia due to insufficient blood and oxygen supply to the heart muscle. Myocardial dysfunction seriously threatens human health and can lead to heart failure, myocardial infarction, and even sudden death [4, 5]. Therefore, real-time analysis of myocardial function is crucial. In recent years, echocardiography has been extensively used in the diagnosis of myocardial dysfunction and the evaluation of viable myocardium due to its advantages of being noninvasive, safe, inexpensive, and simple [6, 7]. Echocardiography can be used to obtain accurate data regarding the cardiac structure and myocardial function and to accurately evaluate the myocardial systolic function and cardiac ventricular wall motion. Therefore, echocardiography has a high application value in diagnosing CVDs and has become an important means for doctor-assisted diagnosis.

Abnormal myocardial function can be evaluated by performing a motion coordination analysis from the echocardiography measurements of the cardiac ventricular wall. Tissue Doppler imaging (TDI) and two-dimensional speckle tracking imaging (2D-STI) are commonly used to evaluate cardiac ventricular wall motion. TDI is an echocardiographic technique used to measure the regional myocardial motion velocity and evaluate the myocardial motion status throughout the cardiac cycle [8], and 2D-STI is a quantitative evaluation method used for tracking the relative position and movement speed of the object of interest in a two-dimensional ultrasound image [9, 10]. Furthermore, myocardial functional coordination can be determined by measuring the time difference to the peak of systolic indicators in different left ventricular segments, which can be obtained using M-mode ultrasound, spectral Doppler, tissue Doppler, speckle tracking, and 3D ultrasound. However, TDI is affected by cardiac rotation, oscillation, and respiratory motion. 2D-STI also has some limitations, such as the requirement of high image quality and high resolution in time and space. If 2D-STI cannot accurately reflect the motion information of all cardiac segments in the same cardiac cycle, it may lead to tracking failure [10]. Therefore, the existing assessment techniques of myocardial wall motion are affected by image quality, which cannot accurately track the myocardial segmental motion and reflect the law of myocardial segmental motion [11].

Segmental motion analysis is commonly used in clinical practice to evaluate the regional left ventricular systolic function more effectively. The 17-segment model recommended by the American Society of Echocardiography [6] is generally used for analyzing the coronary blood supply. However, it is difficult to detect ventricular wall motion in the 17th segment (apical cap), which is the apical region without a heart cavity; thus, the 16-segment model is often used in echocardiography analysis to evaluate the left ventricular wall motion, and the myocardial function is evaluated by analyzing the strain between different segments. The optical flow model is suitable for realizing real-time and high-resolution segmental motion estimation. However, many challenges are encountered in segmental motion estimation when using this method to obtain an accurate dynamic optical flow field. For example, a large amount of target motion is caused by rapid cardiac contraction, and the ordinary optical flow model based on pixel-level operation is unsuitable for large displacements. Furthermore, the image quality of echocardiography is often poor, and the acquisition process is affected by the position of the subject, resulting in a certain degree of “shadow.” The missing information in the “shadow” areas causes estimation errors; blurred echocardiography images also cause large estimation errors.

To quantify the dynamic characteristics of cardiac ventricular wall motion more accurately and evaluate the myocardial function more effectively, we proposed a segment motion analysis method based on multiresolution optical flow tracking in this paper. The proposed method employs multiresolution optical flow estimation, motion vector decomposition, and dynamic tracking of the region of interest (ROI) to accurately calculate the dynamic motion field of each segment and obtain the myocardial segmental motion time curve. The confidence-optimized multiresolution (COM) optical flow calculation method is based on a Gaussian pyramid algorithm and yields high-precision results of the myocardial motion; in addition, confidence weight analysis is employed to improve the accuracy of the optical flow calculation. The echocardiographic images of 25 patients with myocardial dysfunction and 25 healthy volunteers were collected for verification. The results demonstrated that the proposed method is highly accurate, robust, and can be used for myocardial function coordination analysis. Therefore, the proposed method helps improve the quantification of cardiac ventricular wall segmental motion and realize a rapid clinical diagnosis.

## Method

### General framework

**Fig. 1:**
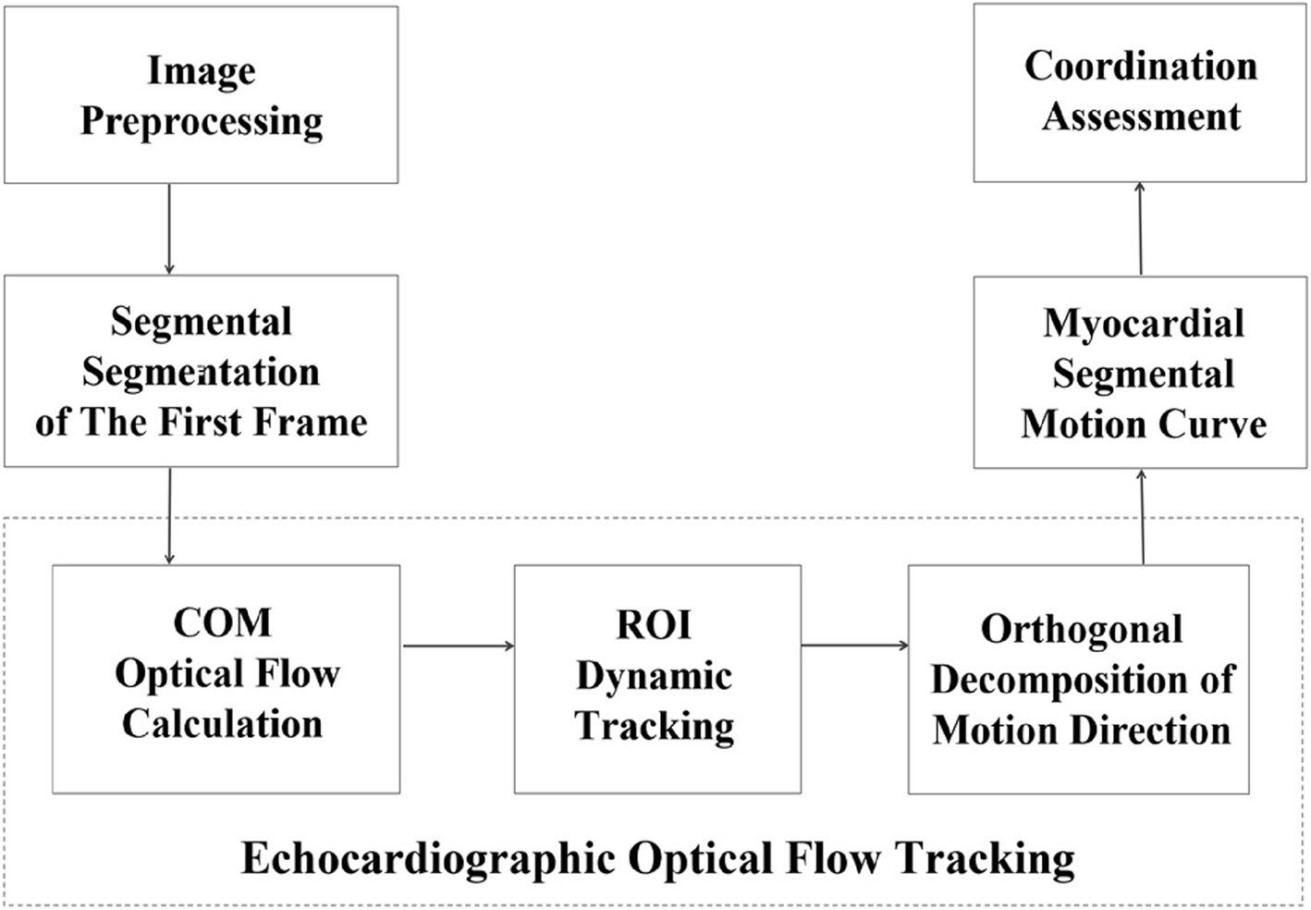
Overall framework of segmental motion estimation for the cardiac ventricular wall

The collected echocardiogram images were used as the original image data, and the sequence of the same section was used as the data input to construct a cardiac ventricular wall segmental motion estimation model based on the optical flow tracking of the echocardiogram. The overall process of the estimation method is illustrated in Fig. 1. First, the contour of the myocardium is manually segmented in the initial frame after the image sequence is preprocessed, and the myocardium segments in different sections are labeled using the 16-segment model recommended by the American Society of Echocardiography. Next, the optical flow model based on COM analysis is used to calculate the cardiac ventricular wall segmental motion displacement distribution. The optical flow field is then calculated for each pair of consecutive frames. Next, the ROI of each segment is dynamically tracked according to the obtained motion field, and the myocardium motion vectors in the region are decomposed into two orthogonal parts: the circumferential vector parallel to the internal myocardium boundary and the normal vector perpendicular to the internal myocardium boundary. Finally, the average displacement and motion curve of each segment are calculated based on the optical flow tracking of the echocardiogram to obtain the motion information of the myocardium segment and facilitate the analysis and evaluation of the coordination of the myocardium segment.

### The COM optical flow calculation

**Fig. 2:**
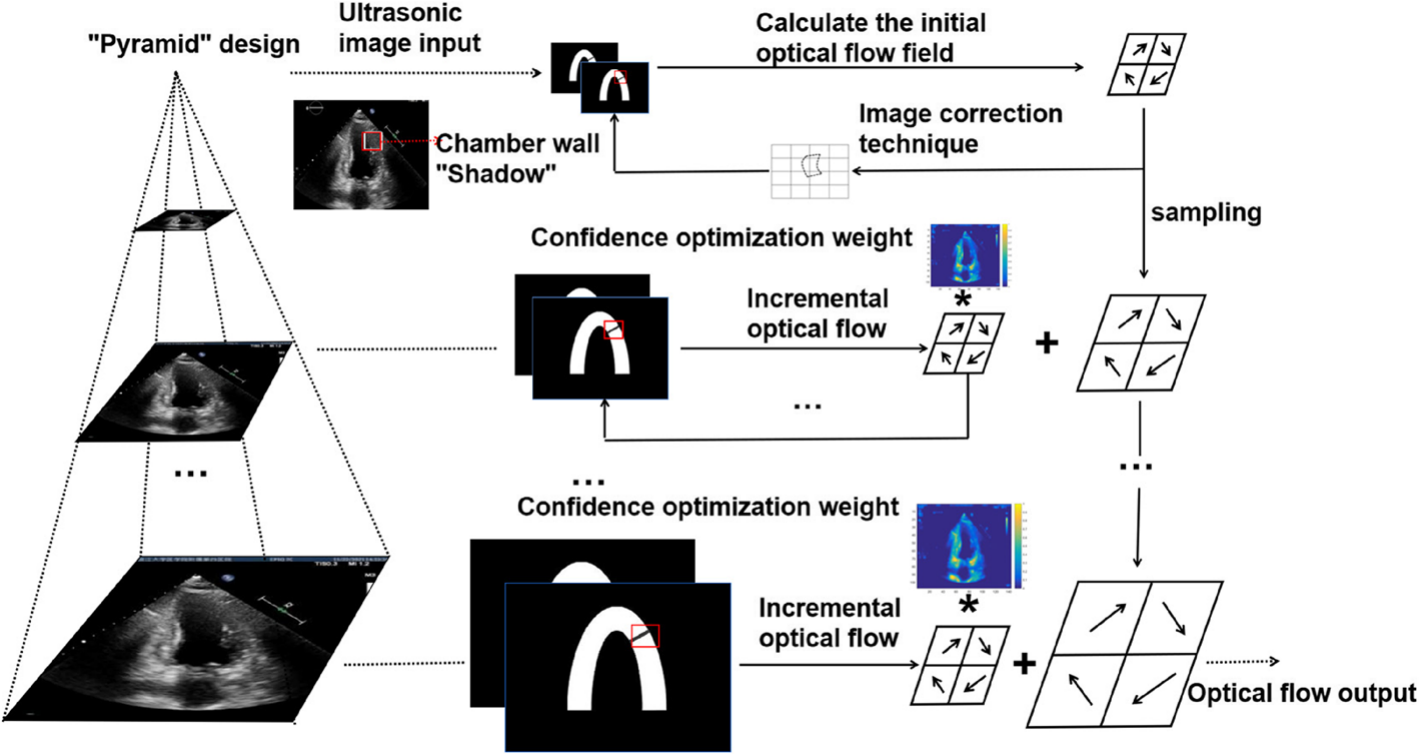
The COM optical flow calculation

The traditional optical flow model is used to calculate the surface luminance pattern for an image [12, 13]. Based on the assumption of constant brightness and continuous time, the smoothness constraint is added to establish the relationship between image pixel motion and gray level. Echocardiographic myocardial motion is large, and echocardiography images contain several artifacts (shadow areas). The traditional optical flow calculation method yields large errors for large displacements and shadow areas. Deep learning algorithms based on neural networks have also been widely used in estimating the optical flow of moving targets in recent years, which can realize adaptive learning of moving targets and improve computation speed and efficiency. However, due to a large amount of training data, data labeling, and operations required, computational efficiency remains low [14–16]. To improve the motion estimation accuracy, the COM optical flow calculation method based on pyramid strategy [17, 18] has been proposed (Fig. 2), wherein two adjacent frames are stratified by different resolutions: large displacements are calculated at a low resolution, and small displacements are calculated at a high resolution. Starting from the top layer of the lowest resolution, the optical flow estimation results of the upper layer are fed back to the next layer as incremental optical flow (corrected motion) for the next layer of images; this process is repeated until the bottom of the pyramid is reached [19–23]. In the process of interlayer transmission of optical flow, image correction technology [17] and confidence optimization screening strategy have been employed: the obtained motion field is used to reverse deform the second frame image to produce a new image that is closer to the first frame, and the new image and the first frame image are transferred to the first layer as image pairs for the incremental calculation of the optical flow field. In addition, the confidence weight matrix is dotted with the incremental optical flow field of this layer and then transferred to the lower layer. The main steps of optical flow calculation for two adjacent frames are as follows:

- Step 1: The initial resolution is selected, the rule that the pyramid of image resolution is gradually increased to the original resolution is determined, and the optical flow field of the image sequence of different resolutions in each layer is obtained. The incremental form of the exponential function is selected for the pyramidal design.
- Step 2: The confidence optimization weight is determined using the input echocardiogram image. The approximation coefficients for wavelet analysis of each layer of the ultrasound image is used as the confidence optimization weight after normalization.
- Step 3: The image of the current layer is used to determine the optical flow field, the optical flow field of the current layer is employed as the incremental optical flow and multiplied with the confidence optimization weight, and then the sampled results of the optical flow field of the previous layer are added to obtain the optical flow field of the current layer.
- Step 4: Step 3 is repeated until the optical flow field under the original resolution (bottom of the pyramid) is iteratively calculated and outputted as the final optical flow field.

**Fig. 3:**
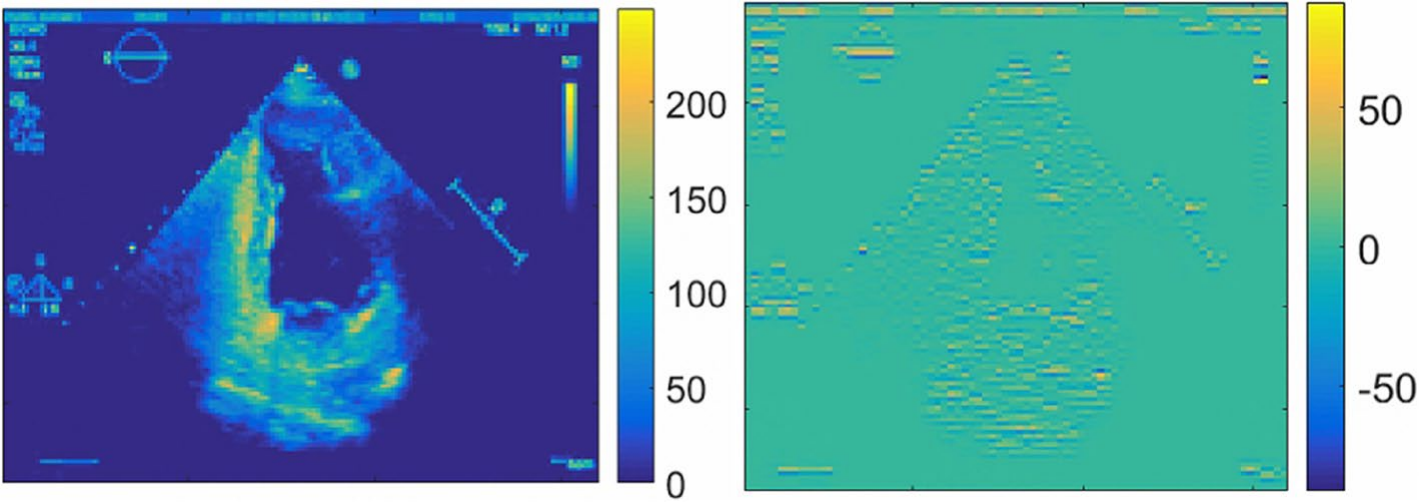
Wavelet decomposition and reconstruction. **a** The first layer of wavelet reconstruction approximation coefficient. **b** The second layer of wavelet reconstruction approximation coefficient

The iterative calculation of the confidence coefficient is vital. The description of the grayscale image is the same as that of the color image. It provides information regarding the distribution and characteristics of the overall and local chromaticity and luminance levels of the image. According to the definition of the optical flow model, the instantaneous change rate of gray level is defined at a specific coordinate point of a two-dimensional image as the optical flow vector, and the gray level of the image reflects the quality of the image. Due to shadow occlusion, the gray level of the image between two frames changes; this affects the optical flow estimation accuracy. Wavelet analysis is a multi-scale and multi-frequency signal processing method that can decompose a signal into scale and frequency components [24]. Wavelet transform has the dual properties of time domain and frequency domain, allowing it to analyze the signal in both time domain and frequency domain at the same time. The signal's effective information is extracted through multi-scale analysis using scaling and shifting operations [25]. Image texture information is extracted using wavelet decomposition and reconstruction, as shown in Fig. 3. Figure 3 shows that the texture information of the original ultrasound image can be retained by the first layer of wavelet reconstruction approximation coefficient. The goal of this paper is to decrease the weight of calculation at low image information while increasing the weight of calculation at rich image texture.

The Gaussian pyramid principle states that the optical flow algorithm can obtain a large rough motion estimate on rough images while fine-tuning the motion to obtain a more accurate motion estimate on fine images. The results normalized by the wavelet approximation coefficient matrix are used as confidence coefficients, which can be added to the iterative optical flow calculation process in Pyramid to avoid directional error in high resolution optical flow calculations. Therefore, the confidence optimization matrix is introduced into the pyramid iteration process, and the incremental coefficient matrix under the corresponding resolution is synchronically updated, yielding a new optical flow field iteration formula, as shown in Eq. (1):

1 $$\left\{\begin{array}{c}u=u+s*du\\ v=v+s*dv\end{array}\right\}$$

where *u* and *v* are the optical flow velocity vectors calculated at the low resolution of the previous layer, *s* is the confidence optimization matrix, and *du* and *dv* are the incremental optical flow fields calculated at the resolution of the latter layer.

The affine technique was used to simulate the motion of myocardial contractions in five cases of echocardiographic data. The left myocardium wall moved 2 pixels to the right, the right myocardium wall moved 2 pixels to the left, and the entire ventricular wall moved 1 pixel down. The calculation range was derived from the myocardial ventricular wall region of interest. The accuracy of the calculated results was compared using different confidence coefficients, and the results are shown in Table 1.

**Table 1:** The results of the calculations using different confidence coefficients

Confidence Coefficient	RMSE	AE
Normalized gray value matrix	0.321	0.947°
Normalized wavelet approximation coefficient matrix	0.301	0.943°

The proposed algorithm computes large motion at lower resolution levels and small motion at higher resolution levels. That is, at a lower resolution, the computed large motion optical flow is used to correct the current frame image, resulting in a similarity between the two frames. The two frames are then synchronized, the size is increased, and the small motion optical flow (incremental optical flow) at a higher resolution is calculated. Therefore, the large-motion optical flow can be added to the incremental optical flow as the output of a specific layer of the pyramid. The "full resolution" optical flow output can be obtained through layer iteration.

### Motion vector decomposition

**Fig. 4:**
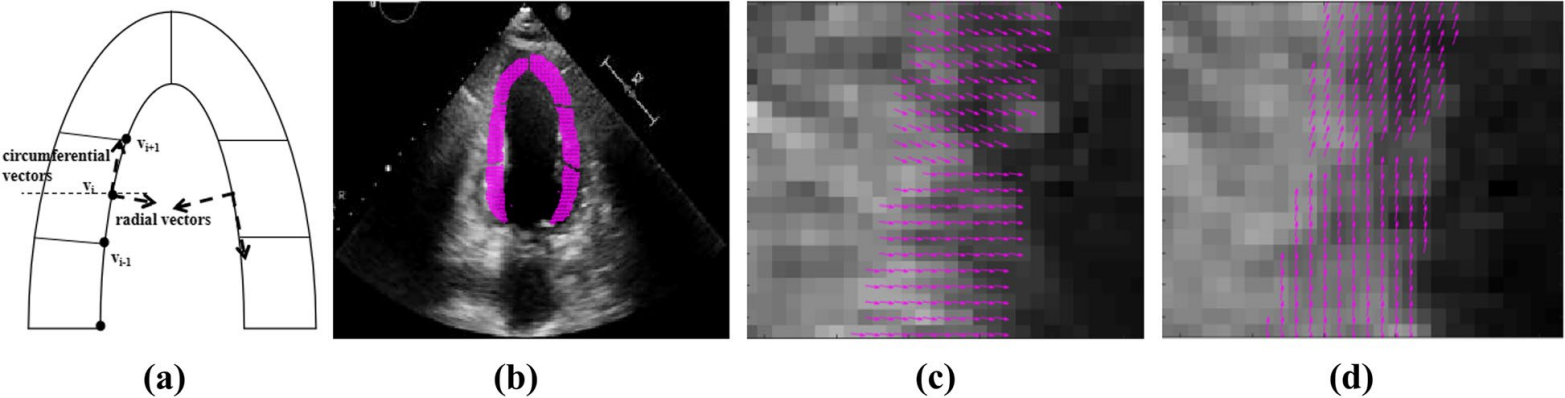
Motion vector decomposition of the cardiac ventricular wall: (**a**) Decomposition; (**b**) ROI vector field; (**c**) Radial normal vector; (**d**) Tangential circumferential vector

The cardiac ventricular wall is shaped like an arch. Under the action of pumping blood, the motion of the cardiac ventricular wall can thus be decomposed into radial and circumferential components that are perpendicular to each other. In this paper, we used a vector separation method to better analyze the motion of the myocardium at different segments of the cardiac ventricular wall. At each point, two orthogonal components are obtained using this method. After the myocardium is manually segmented, the circumferential motion direction of each segment is assumed to be along the ventricular wall. The corresponding normal vector points to the direction of myocardial contraction. Based on the segmented myocardial segments, we connected the two endpoints on one side of the endocardium between each segment, and took the point where the horizontal direction of the midpoint of this line intersects with the endocardium of the segment as the center point of the segment (Fig. 4a). The line connecting the two endpoints of this segment along the clockwise direction of the myocardial wall was taken as circumferential vector for the center point using Eq. (2), where *t*_*i*_ is the circumferential vector of this segment, and *v*_*i*_ is the point at the circumferential vector. The normal vector obtained by rotating it by 90° was taken as radial vector.

2 $${t}_{i}=\frac{{v}_{i}-{v}_{i-1}}{\Vert {v}_{i}-{v}_{i-1}\Vert }+\frac{{v}_{i+1}-{v}_{i}}{\Vert {v}_{i+1}-{v}_{i}\Vert }$$

The motion information contained in the six myocardial segments obtained after manual segmentation can be better observed after the second segmentation. After vector annotation and normalization of the 12 myocardial segments, 12 circumferential and radial normalized motion vectors are obtained. Next, the ROI vector field is obtained from the ROI. The displacement data calculated using the optical flow model are separated using the methods as Eq. (2) to analyze the motion changes caused by the uneven force on the wall boundary. The ROI vector field and its motion vector decomposition are shown in Fig. 4b–d.

### Dynamic tracking of the ROI

**Fig. 5:**
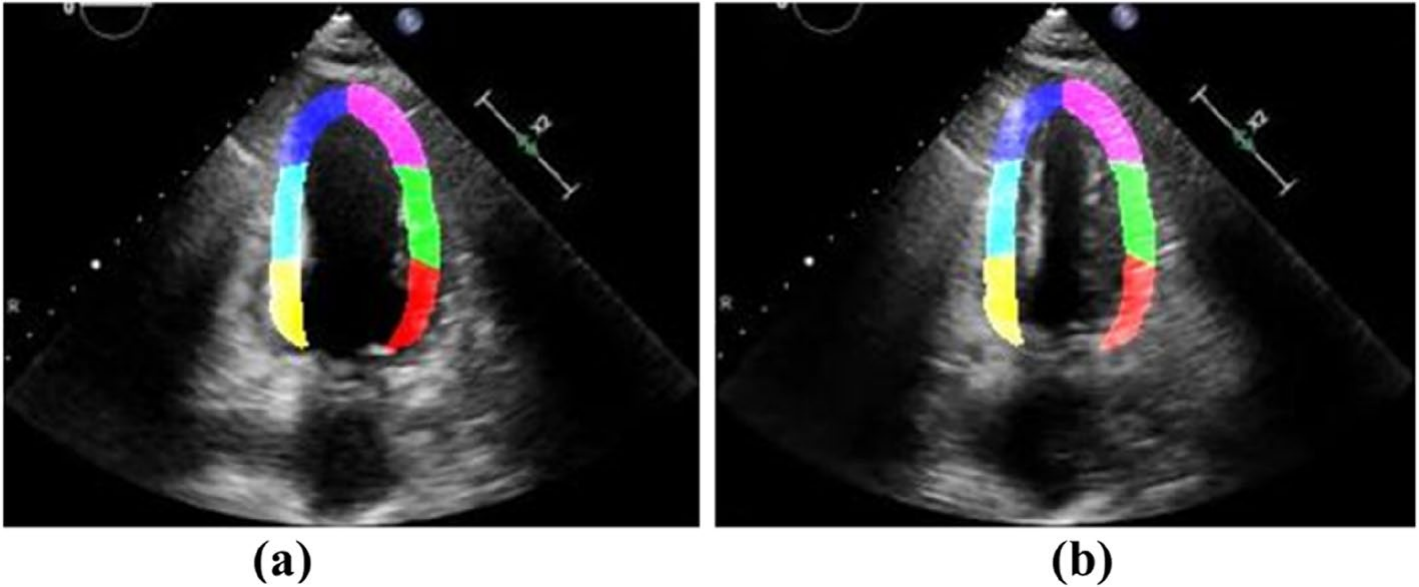
State diagram of the ROI before and after myocardial contraction: (**a**) Initial state of the myocardium; (**b**) State of myocardial contraction

The relative position and shape of the cardiac ventricular wall change greatly during contraction. If the displacement of each segment is always calculated with the myocardial region in the initial state, the actual myocardial position will not match the ROI when the myocardium contracts to a certain state, resulting in large errors in the estimation results of segment displacement, as shown in Fig. 5(a) and (b).

To better track the motion of each segment of the myocardium, we adopted a dynamic tracking algorithm for the ROI of the cardiac ventricular wall to ensure that the ROI is consistent with the actual wall region. The optical flow vector is obtained by performing the optical flow estimation of two image frames. Then the image correction technology is used to map each pixel in the initial ROI *I* to another plane under the action of the optical flow vector to generate a new ROI *I’* and realize the optical flow tracking of the ROI.

**Fig. 6:**
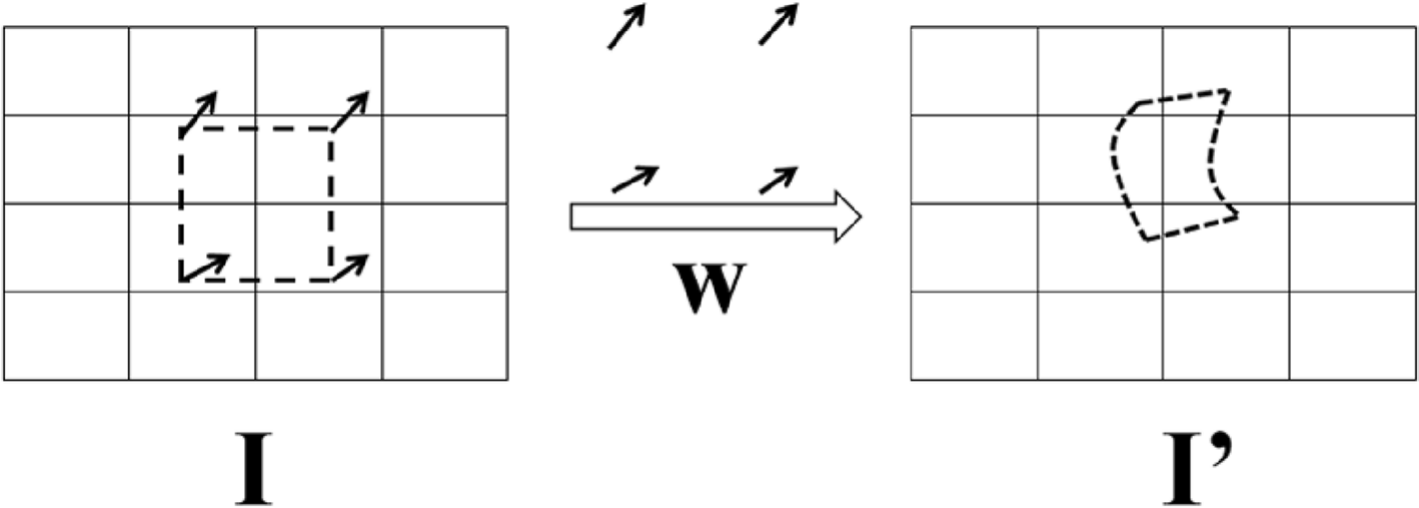
Image correction technology

Pixel correction is at the heart of image correction technology. The motion vector field w is calculated beforehand, and an affine transformation is applied to image I to correct it to a standard scale or angle, so the image I' is obtained. An essential technology for realizing the multi-layer pyramid strategy is image correction technology, which obtains the velocity vector of each pixel after the optical flow calculation of the original image data. Then it generates a new image through reverse deformation of the image, which is compared with the original image to evaluate the algorithm's accuracy. Figure 6 depicts the image correction technique's basic principles. The algorithm can greatly improve optical flow calculation efficiency while tracking fast-moving image pixels.

### Evaluation methods

#### Optical flow error estimation method

The proposed motion evaluation algorithm yields a high accuracy in the velocity vector field computation from the cardiac ventricular wall segmental motion. The root mean square error (RMSE) and angular error (AE) are used as the evaluation index of velocity vector field accuracy. RMSE (Eq. (3)) reflects the dispersion degree between the calculated velocity vector field and the actual velocity vector field [26]. AE (Eq. (4)) reflects the angle error between the calculated optical flow vector and the actual optical flow vector [26, 27]. The calculation range of the optical flow error estimation method is taken from the ROI of the myocardial segments.

3 $$RNSE=\sqrt{\frac1N}\sum\nolimits_i^N\left[\left(U_g^i-U_e^i\right)^2+\left(\left(U_g^i-U_e^i\right)\right)^2\right]$$

4 $${\varnothing }_{E}=\mathrm{a}rc cos\left({v}_{g}-{v}_{e}\right)$$

In Eq. (3), ${\left({u}_{e},{v}_{e}\right)}^{T}$ is the estimated velocity vector field of myocardial movement, ${\left({u}_{g},{v}_{g}\right)}^{T}$ is the actual velocity vector field of myocardial movement, *N* is the number of pixels contained in the ultrasonic echocardiogram, and *i* is the serial number of pixels. In Eq. (4), *v*_*g*_ and *v*_*e*_ are the actual optical flow vector and the calculated optical flow vector, respectively, and *φ*_E_ is the angular error between the calculated optical flow vector and the actual optical flow vector.

#### Estimation method of segmental motion displacement error

The cardiac ventricular wall segmental motion is the main index used to measure whether the myocardial function is abnormal. The strength of the segmental motion, and the amount of displacement are used as the evaluation index [28]. To evaluate the cardiac ventricular wall motion, the displacement error of each segment can be determined using the displacement difference between the ROI and the actual position of the myocardium during the dynamic tracking of the ROI, and the absolute displacement value and relative displacement error under different segments of cardiac ventricular wall motion are calculated. The displacement errors of different segments of the cardiac ventricular wall are quantified to reflect the abnormal motion of the cardiac ventricular wall segment.

**Fig. 7:**
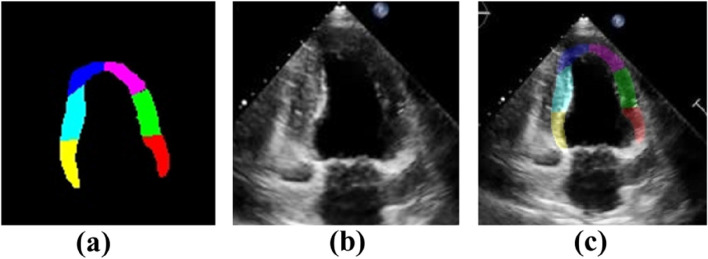
Myocardial images and ROI dynamic tracking experiment: (**a**) Segmented ROI; (**b**) Myocardial image data; (**c**) Results after superposition of the two

**Fig. 8:**
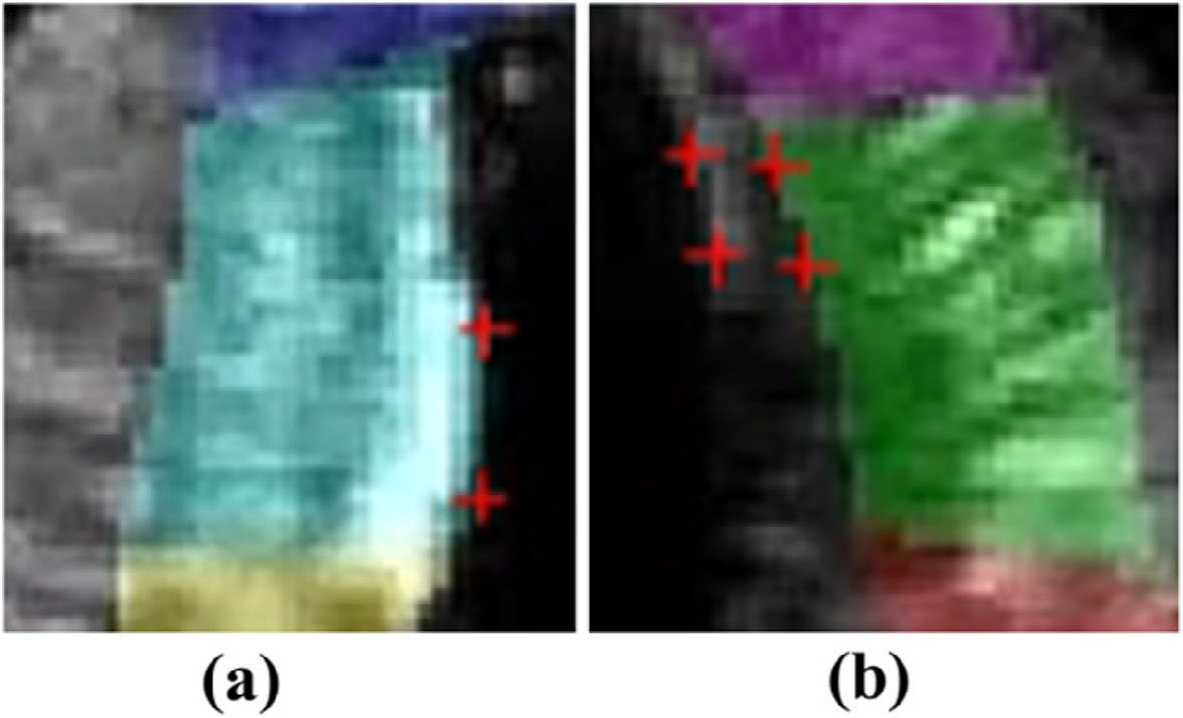
ROI displacement error analysis method of cardiac ventricular wall segment: (**a**) In the case of no error; (**b**) In the case of error

The quantitative assessment of the cardiac ventricular wall segmental motion is highly significant in analyzing whether myocardial motion is abnormal. We experimentally verified the accuracy of the proposed method by using the displacement error. In total, 50 clinical cases (25 patients with myocardial dysfunction and 25 healthy volunteers) were selected for the experiment, as shown in Fig. 7. Displacement error analysis was performed using the displacement difference between the ROI and the actual position of the myocardium during the dynamic tracking of the ROI, as shown in Fig. 8. The absolute displacement values and relative displacement errors under different segments of cardiac ventricular wall motion were calculated; the results are presented in Table 3.

## Experiment

### Experimental data

Color Doppler echocardiography images of 50 clinical cases (25 patients with myocardial motion abnormalities and 25 healthy volunteers) collected during January 2021–December 2021 were used as the experimental data. All imaging data were acquired in the bedside ultrasound (Philips EPIQ 7C cardiovascular ultrasound imaging device) with X5–1 probe and a frequency range of 1.0–5.0 MHz. The image dataset had a uniform frequency of 50 Hz, a temporal resolution of 0.02 s, a spatial resolution ranging from 1.3 to 2.6, and a frame rate of 50 frames per second. The image dataset used in this paper has a frame number that corresponds to the number of image frames in one cardiac contraction cycle, which is typically 9 to 13 frames. The patients were placed in the left decubitus position, and the probe was placed on the chest (between the second and fifth ribs) to observe the long axis of the left ventricle, two apical chambers, four apical chambers, and multiple short axis sections of the left ventricle. The myocardium wall motion was observed to check whether there was abnormal motion, focusing on the observation of the two chambers of the left ventricle. Ultrasonic echocardiograms of the left ventricle of patients were collected to analyze the cardiac ventricular wall segmental motion. The proposed true value of myocardial motion as simulated by affine transformation is referred to as the ground truth.

### Myocardial motion

**Fig. 9:**
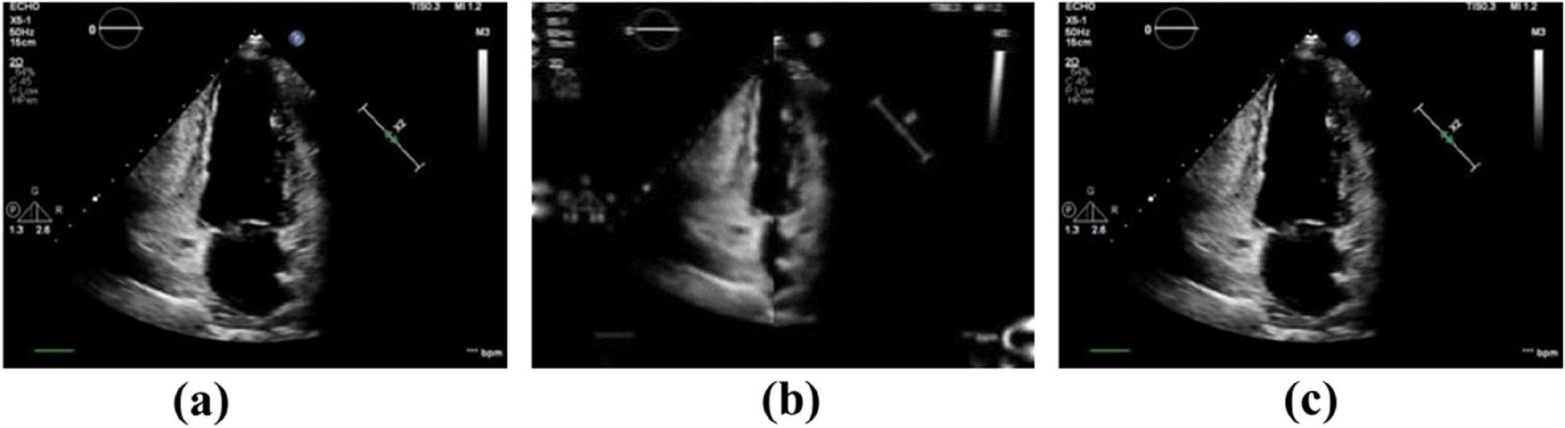
State diagram of cardiac ventricular wall motion: (**a**) Original myocardial state; (**b**) State of myocardial contraction; (**c**) State of myocardial relaxation

We estimated the segmental motion of the cardiac ventricular wall. In one cardiac cycle, motion vector decomposition was performed on the motion process of the cardiac ventricular wall from relaxation to contraction, and the ROI of the cardiac segments was dynamically tracked to better track the motion state of cardiac segments. The process of the left cardiac ventricular wall motion is illustrated in Fig. 9.

### Segmentation

**Fig. 10:**
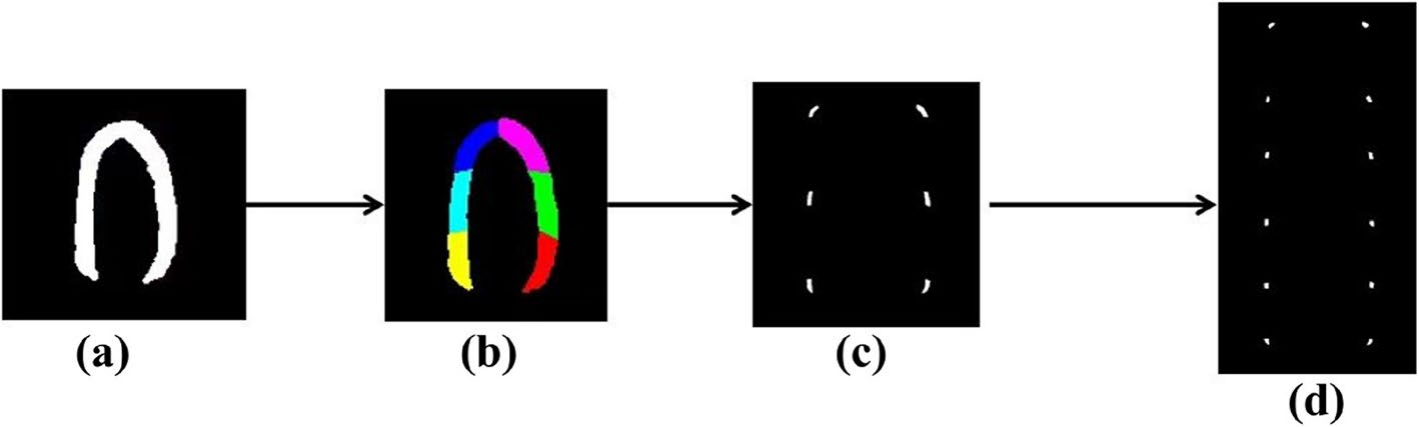
Segmentation of cardiac ventricular wall segments: (**a**) Myocardium contour; (**b**) Myocardial segmentation model; (**c**) Six segments of the Myocardium; (**d**) Twelve segments of the myocardium

In this study, 25 patients with myocardial dysfunction were selected as the study group, and 25 healthy volunteers were selected as the control group. The systolic period of cardiac ventricular wall motion was recorded, the radial displacement curve of cardiac segments was drawn, and the segmental motion was evaluated. The myocardium wall of the left ventricular two-chamber heart was manually segmented, first determining the points at the apex and base of the heart, and then measuring the side lengths of the apex and base. Finally, they were divided into three sections. Therefore, six segments were identified. One experienced sonographer manually segmented the myocardial contour, which was then verified by another sonographer of the same level. When the two sonographers' segmentation results were inconsistent, a third sonographer was invited to correct the segmentation results, and the revised segmentation results were considered the final results. In this study, myocardial segments were labeled using the American Society of Echocardiography's 16-segment model. A total of 6 segments of the apical two-chamber view of the left ventricular were focused on myocardial wall motion. In this paper, 12 segments were obtained after secondary segmentation to better observe the motion information within the 6 segments. The segmentation process of cardiac ventricular wall segments is depicted in Fig. 10.

## Results

### Segmental motion curves

**Fig. 11:**
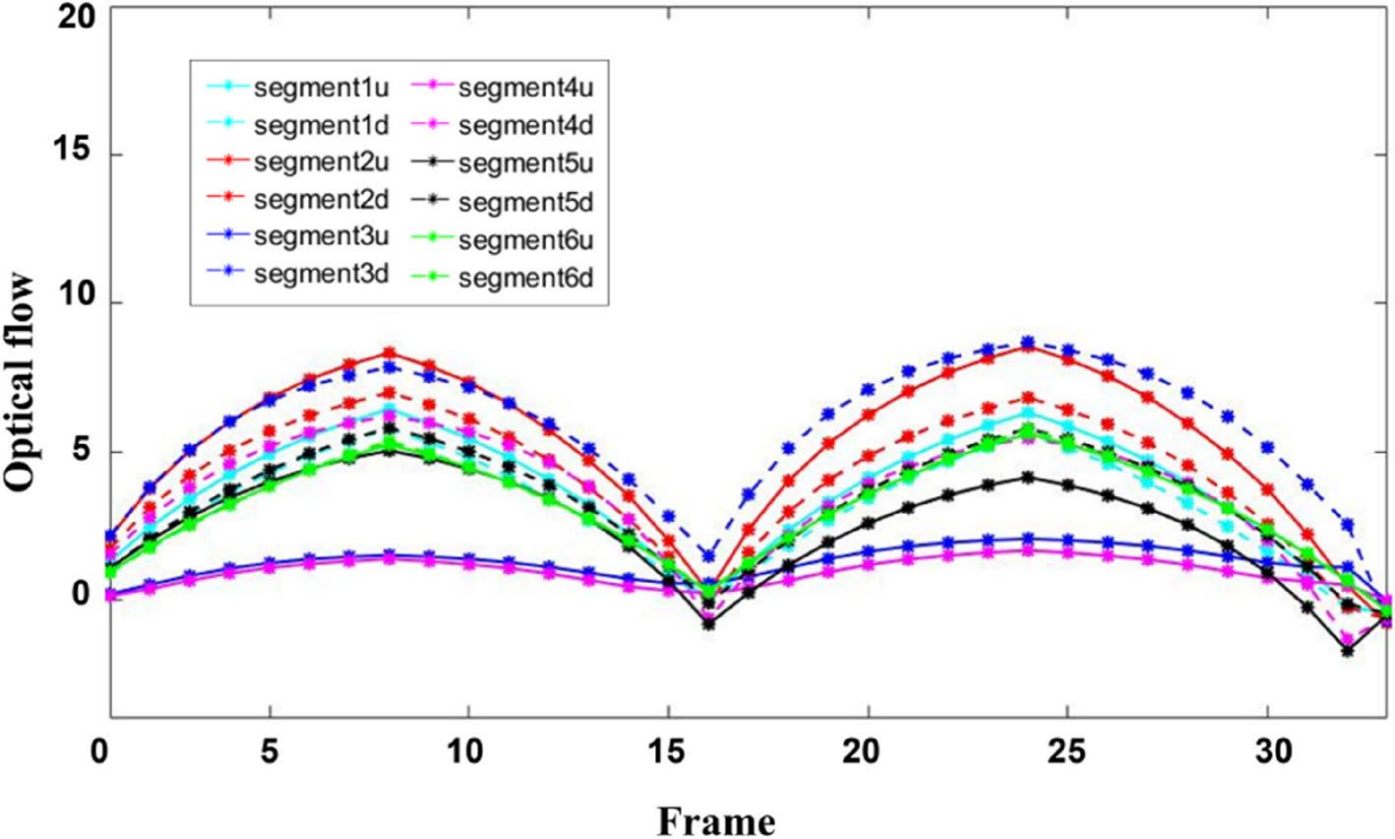
Radial displacement curve of cardiac ventricular wall motion

The whole process of myocardial wall contraction and relaxation was simulated twice by image correction technique, and the displacement curves of each segment were obtained by calculating the mean displacement of pixels in the ROI of each segment of the myocardium, as shown in Fig. 11.. The 12 curves in Fig. 11 represent the radial displacement of each segment after direction separation. This curve reflects the overall displacement of each myocardial segment and can be used to obtain the motion details inside the segment. The displacement curves drawn in the two cardiac cycles exhibited regular arches, which proves the accuracy of the proposed method.

**Fig. 12:**
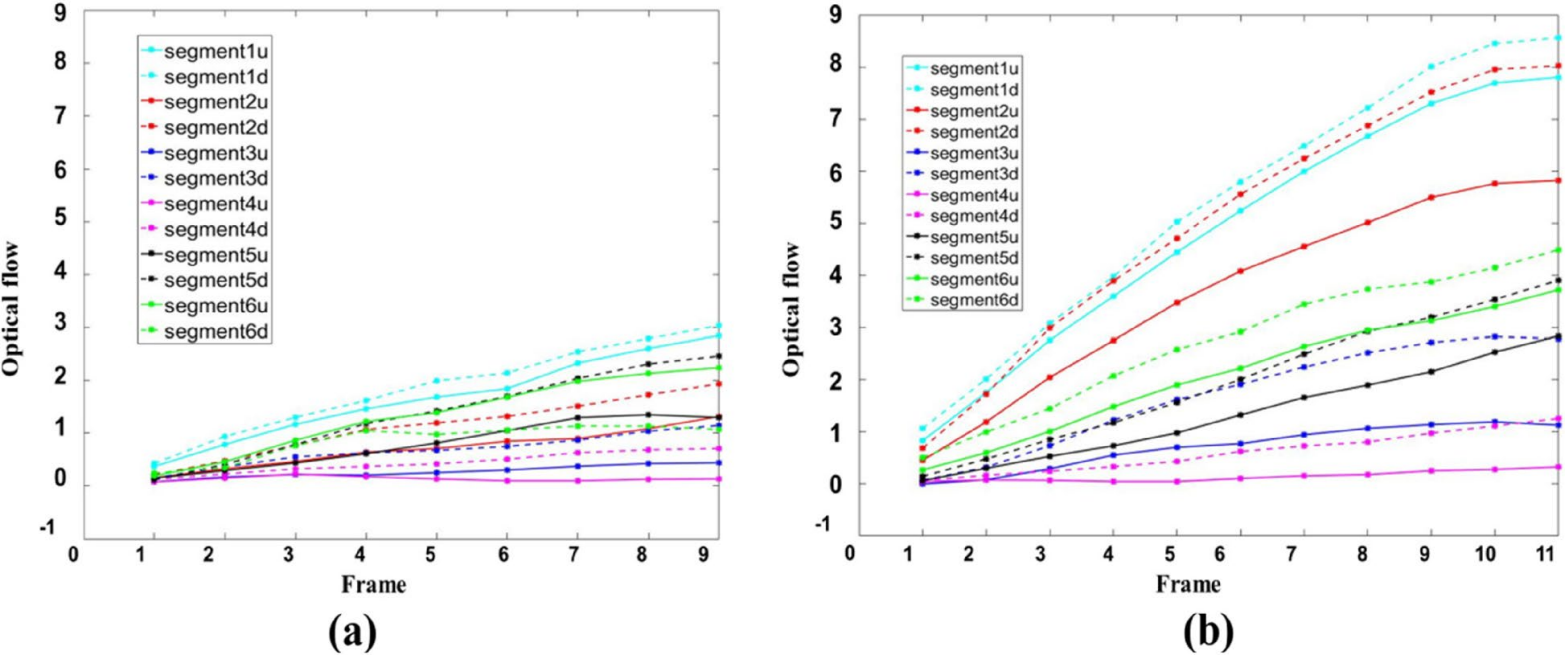
Radial displacement curve during systolic cardiac ventricular wall motion: (**a**) Radial displacement curve of patients with abnormal myocardium; (**b**) Radial displacement curve of healthy volunteers

Individual data of healthy volunteers and patients with myocardial abnormalities in the image dataset of this paper were selected for display. Figure 12 (a) and (b) show the curve of myocardial motion in patients with myocardial dysfunction and healthy volunteers, respectively. After the direction separation of each segment, the motion amplitude of each segment in patients with myocardial dyskinesia was smaller than that of healthy volunteers. It belonged to the weakened motion of the cardiac ventricular wall. Thus, the segmental motion curve can assist in detecting abnormal cardiac ventricular wall motion.

### Comparison of optical flow errors

We validated the proposed method's effectiveness and accuracy by comparing it to the Horn-Schunck(HS) optical flow model, Lucas-Kanade(LK) optical flow model, and Brox optical flow model using two indexes, RMSE and AE; the results are shown in Table 2. The parameters of the HS and LK optical flow models were used without tuning in the original paper. The proposed method and the Brox optical flow method are used to tune the parameters, and the RMSE error experiment is used to adjust the relaxation parameters. Furthermore, the proposed method improves the optical flow calculation process starting with the initial resolution selection and confidence coefficient.

**Table 2:** Experimental results of the proposed algorithm, HS optical flow model, LK optical flow model, and Brox optical flow model

Algorithm	Root mean square error (RMSE)	Angular error (AE)
HS optical flow	0.960	7.133°
LK optical flow	0.854	11.404°
Brox optical flow	0.532	7.802°
Proposed algorithm	0.257	3.587°

The proposed method yielded the lowest RMSE and AE values among all the optical flow models studied herein, thus, indicating that the proposed method has the highest computational accuracy.

### Comparison of segmental motion displacement errors

**Fig. 13:**
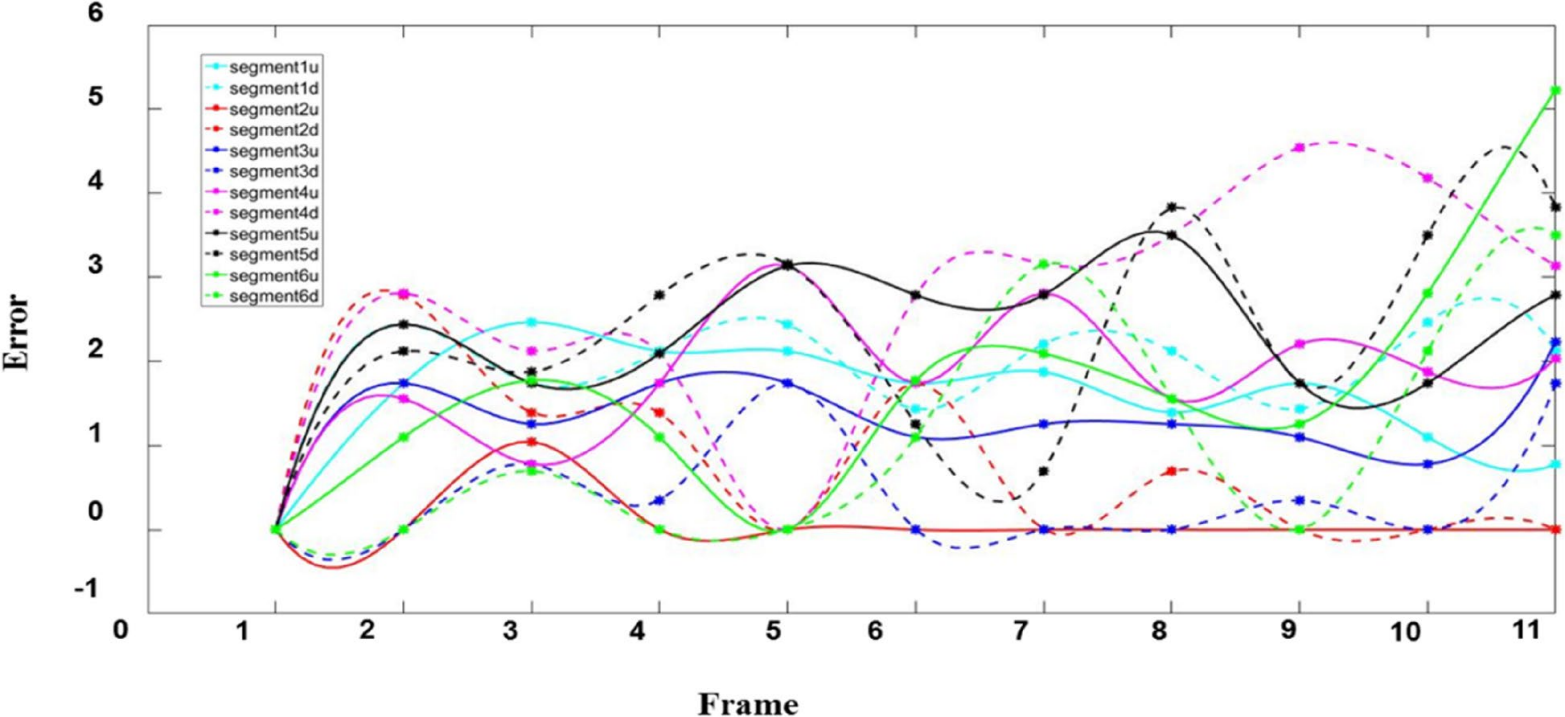
Radial displacement error curves of 12 segments of cardiac ventricular wall motion

We compared the segmental motion displacement errors in the patients with myocardial dysfunction with those in the healthy volunteers, as shown in Fig. 12. Displacement error analysis was performed using the displacement difference between the ROI and the actual position of the myocardium during the dynamic tracking of the ROI, as shown in Fig. 13. The average values of absolute displacement and relative displacement error of myocardial wall motion in different segments of all healthy volunteers and patients with abnormal myocardial motion were calculated (Table 3).

**Table 3:** Absolute displacements and relative displacement errors in different segments of cardiac ventricular wall motion

Heart sarcomere section	Study group of patients with myocardial dysfunction		A control group of healthy volunteers	
	Displacement values (mm)	Error (mm)	Displacement values (mm)	Error (mm)
Segment 1 upper end	2.857	1.112	3.565	1.124
Segment 1 lower end	1.638	1.215	2.345	1.221
Segment 2 top end	2.359	0.985	4.862	0.676
Segment 2 lower end	2.954	1.123	5.145	0.887
Segment 3 top end	0.745	2.054	1.314	1.421
Segment 3 lower end	1.349	1.456	2.945	0.965
Segment 4 top end	0.356	2.415	0.314	2.129
Segment 4 lower end	0.845	2.211	1.121	2.128
Segment 5 upper end	1.125	1.856	2.326	2.459
Segment 5 lower end	1.765	1.967	3.485	2.125
Segment 6 upper end	2.157	1.926	3.855	2.015
Segment 6 lower end	1.795	1.846	4.247	1.385

### Comparison of optical flow computational efficiency

The calculation time of optical flow in each frame of an echocardiogram was compared in this paper to assess the computational efficiency of different algorithms, and the results are shown in Table 4. Table 4 shows that the proposed algorithm has a higher computational efficiency than the Brox optical flow method, but it takes longer to compute than the HS optical flow method and the LK optical flow method. This is because the HS optical flow method and the LK optical flow method do not employ a pyramid iteration strategy, resulting in a higher computational efficiency. In this paper, the number of pyramid iterations is reduced in comparison to the Brox optical flow method, improving computational efficiency.

**Table 4:** Optical flow computational efficiency comparison results

Algorithm	computation time(s)
HS optical flow	2.525 s
LK optical flow	4.902 s
Brox optical flow	10.576 s
Proposed algorithm	9.007 s

## Discussion

In the proposed method, multiresolution optical flow estimation is used to improve the estimation accuracy of the cardiac ventricular wall motion, and ROI dynamic tracking is used to ensure the accuracy of the motion curve of each segment. Next, the cardiac ventricular wall motion is decomposed into vectors, and the radial vector consistent with the motion direction of the cardiac ventricular wall is selected for analysis. Finally, ROI dynamic tracking is performed to accurately calculate the motion characteristics of the myocardial segments. Simulation results demonstrated that the proposed method could improve the optical flow estimation accuracy. Furthermore, the proposed method was used to draw the myocardial segment displacement curve and calculate the absolute displacement and relative displacement error under different segments of myocardial wall motion using actual data from patients with myocardial dysfunction and healthy volunteers. The results demonstrated that the estimation results obtained with the proposed method are robust. The RMSE and AE, as well as the absolute and relative displacement errors under different segments of cardiac ventricular wall segmental motion, were used to validate the proposed method's accuracy. The proposed method resulted in the smallest RMSE and AE. Therefore, the proposed method can track a moving object quickly and can overcome the estimation error caused by poor image quality.

### Shadowing

**Fig. 14:**
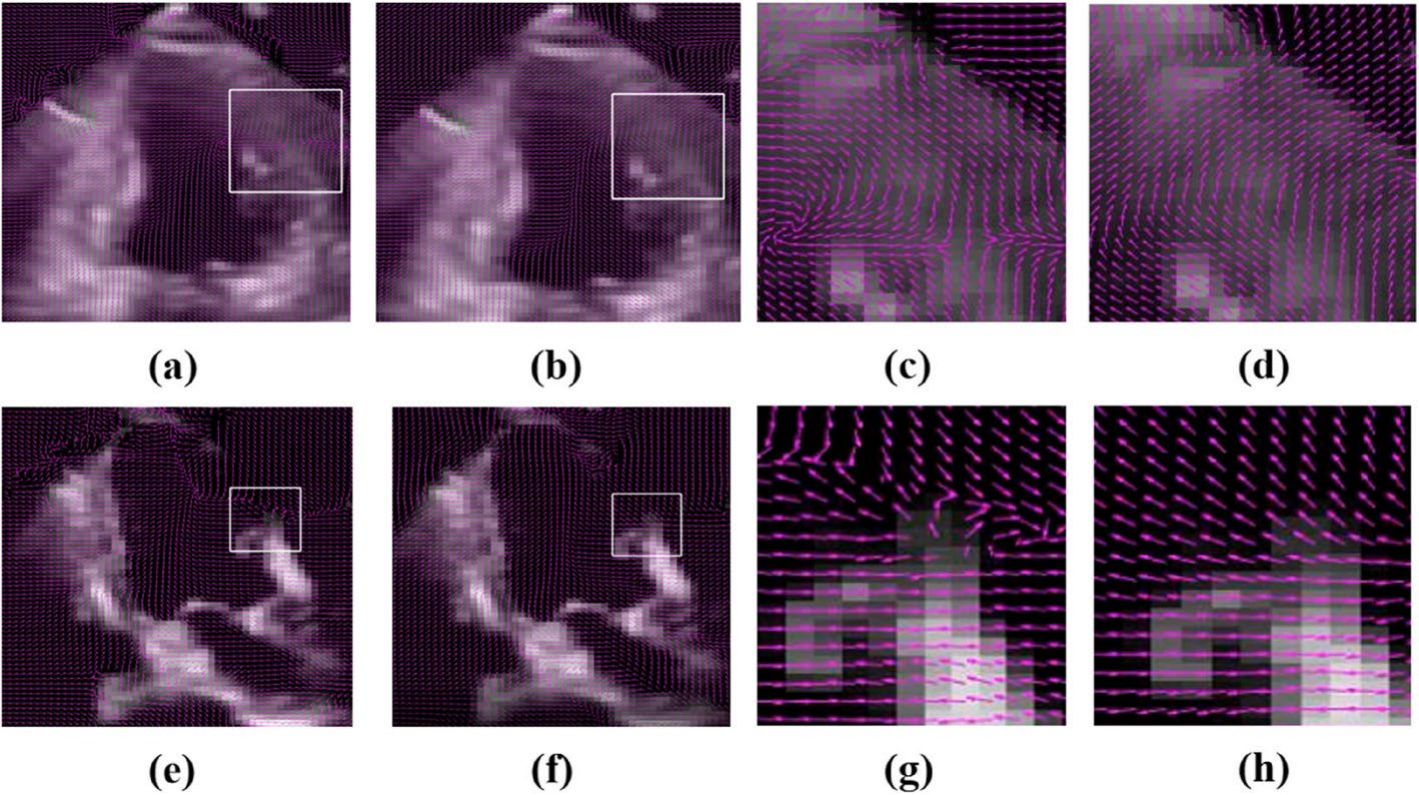
Estimation results of optical flow at local shadow areas: (**a**, **c**, **e**) and (**g**) Results without confidence optimization; (**b**, **d**, **f**) and (**h**) Results after confidence optimization

During echocardiography, because the clinician typically captures the ultrasound image in the patient’s left decubitus position, rib occlusion often creates shadowing in the echocardiographic data. At low resolutions, the size of the shadow is relatively small, and the corresponding movement of the shadow can better reflect the overall movement of the myocardium in the region. At high resolutions, the calculation of the area with obvious brightness is more accurate. In contrast, the calculation of the shadow area leads to errors in optical flow estimation due to the lack of information. In this paper, we proposed a confidence optimization method based on wavelet analysis to reduce the shadow effect at high resolutions while retaining the optical flow distribution at low resolutions. The influence of the shadow area on optical flow estimation is reduced after multiresolution optical flow estimation. As shown in Fig. 14, we chose echocardiograms with local shadows and compared the results obtained using the proposed method to those obtained using the conventional method. After the addition of the wavelet approximation coefficient matrix, the weight of shadows in the iterative process was relatively small, which reduced estimation errors caused by missing information at high resolutions and solved the problem caused by shadow areas.

**Fig. 15:**
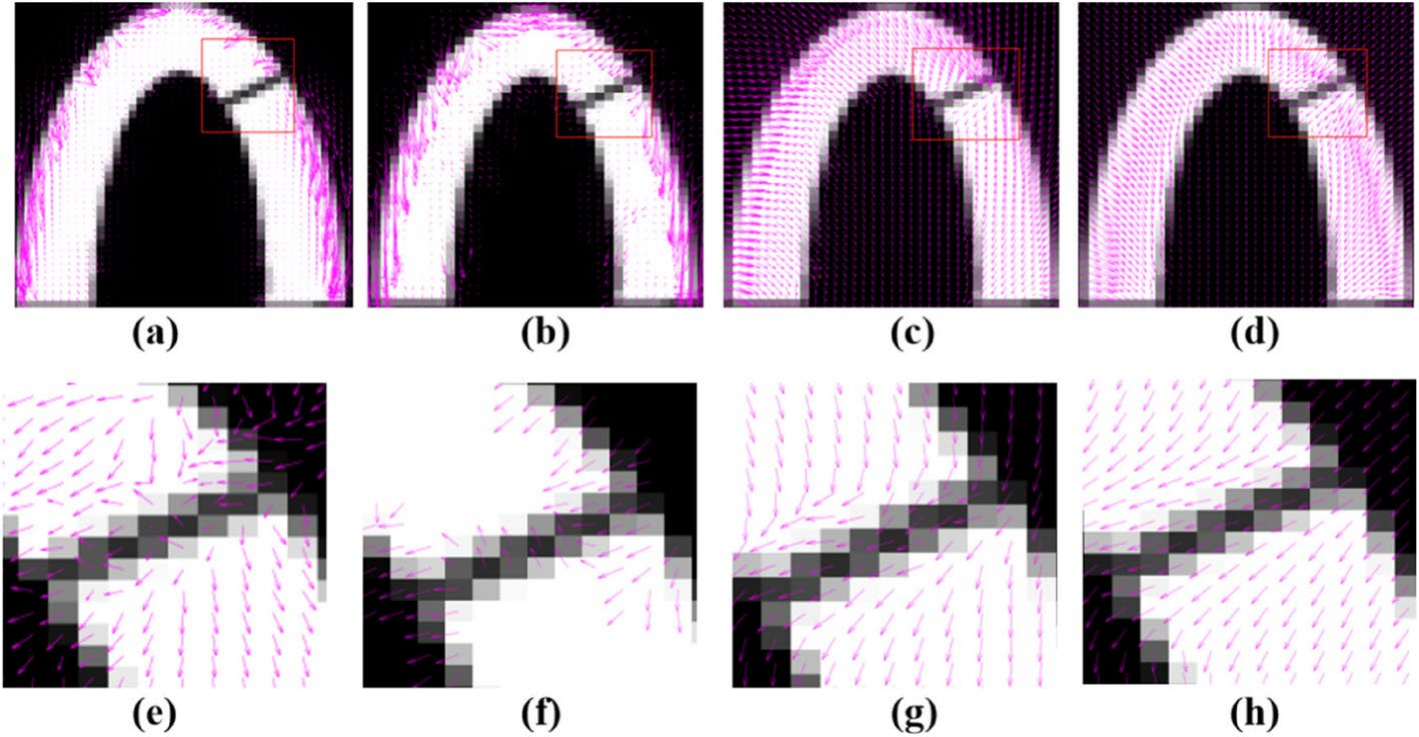
Optical flow field of cardiac ventricular wall simulation image with a shadow area added and optical flow distribution near the shadow area: (**a**)and (**e**) represent the HS optical flow model; (**b**) and (**f**) represent the LK optical flow model; (**c**) and (**g**) represent the Brox optical flow model; (**d**) and (**h**) represent the proposed method

To simulate the effect of myocardial motion, we simulated a U-shaped binary image and added a shadow to the right side of the ventricular wall to simulate myocardial contractile motion by using the warp operation. The left side of the simulated ventricular wall moved 2 pixels to the right, the right side of the ventricular wall moved 2 pixels to the left, and the entire ventricular wall moved 1 pixel downward. The optical flow field of simulated data for the cardiac ventricular wall with shadows was calculated using the HS optical flow model, the LK optical flow model, the Brox optical flow model, and the proposed method (Fig. 15). It could see that the error rate was high for the HS optical flow model and LK optical flow model. Moreover, the HS optical flow model, LK optical flow model, and Brox optical flow model all show orientation errors in the calculation of the "shadow" and the manual segmentation of the ROI. The results of the proposed method were superior to the other three methods. It can reduce the calculation error caused by missing information at high resolution, effectively solve the problem of motion estimation error caused by shadows and thus yield improved estimation accuracy.

### ROI dynamic tracking

When drawing the motion curve, the displacement of each segment represents the mean displacement of all pixels in the ROI corresponding to this segment. The actual displacement of each segment was estimated from the initial segmented image at the start of the experiment; however, as the myocardium contracted, the actual position of each segment changed significantly from the initial position, resulting in errors in the final drawn displacement curve. Therefore, in this paper, we proposed a dynamic ROI tracking method. To ensure high accuracy in the final segment displacement estimation, we used the warp operation to track the initial segmented images in real time. We used the normal ultrasonic echocardiogram data of a healthy volunteer to capture the process of myocardium from diastole to contraction in one cardiac cycle. The ROI dynamic region tracking algorithm was used to track the entire cardiac ventricular wall, which can track the motion state of each segment of the myocardium well; thus, improving the estimation accuracy of cardiac ventricular wall segmental motion.

### Resolution

To improve the estimation accuracy of the optical flow rate of cardiac ventricular wall segmental motion, we adopted a multiresolution analysis method to reduce the error; this approach requires selecting the original images to start the optical flow estimation after reducing them to different scale sizes. The experiment used 20 cases of cardiac hyperimage data to determine the optimal top pyramid size. The optical flow field was set to deform the image using image reverse warp operation to obtain the second image after deformation. The proposed optical flow model was then used to calculate the optical flow of cardiac ventricular wall motion between the two frames, and the error between the set optical flow field and the calculated optical flow field was compared at different reduced sizes. The experimental comparison results revealed that, in most cases, the error was the smallest when 30% of the original image was selected.

In the clinical ultrasound image data acquisition process, the relative position of the myocardium in the process of contraction and diastole changes due to the movement of the probe position by the doctor and the change in the patient’s position, resulting in errors in segmental motion estimation. Thus, it is necessary to further improve the image position correction to reduce the error. In future research, more cardiac cross-sections (such as the long axis of the left ventricle, four apical chambers, and multiple short axes of the left ventricle) need to be studied to calculate the myocardial segmental motion to improve the generalizability of the proposed algorithm and for its extensive application in clinical practice.

Our research has some limitations in terms of manual segmentation and ROI dynamic tracking. Currently, selecting the cardiac ventricular wall requires manual segmentation, which takes a significant amount of time and effort on the part of clinicians. Therefore, automatic intelligent segmentation is required to reduce clinician workload. Furthermore, the accuracy of optical flow calculation affects ROI dynamic tracking. If the image quality is too poor, optical flow calculation errors will accumulate, resulting in a large difference between the final result and the true value.

## Conclusions

To solve the problems that ultrasonic echocardiogram is heavily dependent on the physician’s subjective judgment and the large workload of myocardial motion abnormality identification, we proposed a method based on the optical flow tracking of echocardiogram by using multiresolution optical flow estimation, motion vector decomposition, and ROI dynamic tracking. In the proposed method, first, the cardiac ventricular wall contour is manually segmented, and myocardial segments are marked. Following that, the motion parameters of the cardiac ventricular wall segmental motion are computed using multiresolution optical flow estimation. Finally, to accurately reflect the motion information of the myocardial segments, the displacement data of each myocardial segment are calculated using motion vector decomposition and ROI dynamic tracking. The results demonstrated that the proposed method could effectively solve the problem of estimation errors caused by shadowing, improve the estimation accuracy of motion displacement, and can quantify cardiac ventricular wall segmental motion for the quantitative assessment of segmental motion abnormalities and assist in ultrasound monitoring or diagnosis.

To provide clinicians with a foundation for quantitative evaluation, the results of healthy and unhealthy patients can be qualitatively analyzed in conjunction with the quantitative analysis results of the algorithm in this paper. The segmental motion of the myocardial wall was clinically graded into different ranges. If the average displacement of the ventricular wall segments was greater than 5 mm, it was classified as grade 0, indicating normal or hyperactive ventricular wall motion. If the average displacement of the ventricular wall segments was between 2 and 5 mm, it was classified as grade 1, indicating reduced wall motion. Grade 2 was defined as the disappearance of ventricular wall motion if the average displacement of the segments of the ventricular wall was between 0 and 2 mm. The rest were grade 3 anomalous wall motion or paradoxical motion. The displacement calculation error of segment 2 is small in this experiment, and the reference value is strong. Segments 3 and 4 have a small displacement in the assumed motion direction, and the reference value is low. The remaining segments can serve as supplemental evaluation methods. In the future, the algorithm could be used in clinical practice. The grading of the displacement value can help clinicians conduct qualitative analysis to determine whether the patient has a myocardial abnormality and the severity of the abnormality.

According to sonographers' and clinicians' experience in bedside operation, in the daily diagnosis and treatment of ICU patients, most mechanical ventilation patients have no significant effect on the stability of image acquisition, with the exception of a few terminal or severe patients who require large parameters to assisted ventilation. Stable images can be obtained in this paper, and the algorithm can assist non-experienced ultrasound medical workers in identifying abnormal myocardial motion.

## Data Availability

The datasets generated and analyzed during the current study are not publicly available due to information that could compromise the privacy of research participants but are available from the corresponding author at reasonable request.

## References

[1] World Health Organization (2021). Cardiovascular diseases (CVDs).

[2] Release of the China Cardiovascular Health and Disease Report 2021:2 out of every 5 deaths are due to cardiovascular disease.Chinadaily.com.cn [quoted on 2022–06–24].

[3] World Health Organization (2020). The Top 10 Causes of Death.

[4] Peng R, Peng J (2018). Application and progress of echocardiography in evaluation of coronary heart disease. Prog Cardiol.

[5] Yuwei S, Ling J (2021). Clinical application of stress echocardiography in ischemic heart disease. Prog Cardiol.

[6] Clinical application guideline of echocardiography in evaluating systolic and diastolic function of heart. Chin J ultrasound imaging, 2020;29(06):461–477.

[7] Lang RM, Badano LP, Mor-Avi V (2015). Recommendations for Cardiac Chamber Quantification by Echocardiography in Adults: An Update from the American Society of Echocardiography and the European Association of Cardiovascular Imaging. Eur Heart J Cardiovasc Imaging.

[8] Pernot M , Villemain O . Myocardial Stiffness Assessment by Ultrasound: Are We Ready for the Clinical "Lift Off"?. . JACC. Cardiovasc Imaging, 2020;13(11). 10.1016/j.jcmg.2020.07.024 33008759

[9] Wu D, Li P, Tian HY (2019). Evaluation of left ventricular systolic function by two-dimensional speckle tracking imaging. China Med Rev.

[10] Yu HK, Lin H, Wang H (2009). Evaluation of right ventricular function by echocardiography. J Clin Ultrasound Med.

[11] Katikireddy CK, Acharya T (2018). Myocardial segmental thickness variability on echocardiography is a highly sensitive and specific marker to distinguish ischemic and non-ischemic dilated cardiomyopathy in new onset heart failure. Int J Cardiovasc Imaging.

[12] Horn BKP, Schunck BG (1981). Determining optical flow. Artif Intell.

[13] Shao XQ, Yang Y, Liu YL (2021). Review of optical flow algorithms in fluid motion estimation. J Image Graph.

[14] Dosovitskiy A, Fischery P, Ilg  E (2015). Flownet: learning optical flow with convolutional networks. Proceeding of 2015 IEEE International Conference on Computer Vision.

[15] Ilg E, Mayer N, Saikia T (2017). Flownet 2.0: evolution of optical flow estimation with deep networks. Proceeding of 2017 IEEE Conference on Computer Vision and Pattern Recognition.

[16] Zhenglai W, Min H, Qibing Z, Sheng J (2018). The optical flow detection method of moving target using deep convolution neural network. Opto-Electron Eng.

[17] Brox T, Papenberg N, Weickert J (2004). High accuracy optical flow estimation based on a theory for warping. Proc 8th Eur Conf Comput Vis.

[18] Papenberg N, Bruhn A, Brox T (2006). Highly accurate optic flow computation with theoretically justified warping. Int J Comput Vision.

[19] Heitz D, Mémin É, Schnörr C (2010). Variational fluid flow measurements from image sequences: synopsis and perspectives. Exp Fluids.

[20] BAI J, Huang Ll (2018). Research on LK Optical Flow ALgo-RIthM with Gaussian Pyramid Model Based on Open CV ForSingle Target Tracking. 2018 2nd International Confer-ence on Artificial Intelligence Applications and Technolo-Gies.

[21] Liu K, Wei SX, Chen ZJ (2017). A real-time high per-formance computation architecture for multiple moving tar-get tracking based on wide-area motion Imagery via Cloudand Graphic Processing Units. Sensors.

[22] Mémin É, Pérez P (1998). Dense estimation and object-based segmentation of the optical flow with robust techniques. IEEE Trans Image Proc.

[23] Zhou L, Kambhamettu C, Goldgof DB (2000). Fluid structure and motion analysis from multi-spectrum 2D cloud image sequences. Proceedings of 2000 IEEE Conference on Computer Vision and Pattern Recognition. Hilton Head Island.

[24] Guo TT, Zhang TP, Lim E (2022). A review of wavelet analysis and its applications: challenges and opportunities[J]. IEEE Access.

[25] Wang R, Zhu Q, Bu WC (2022). Multimedia image data compression based on wavelet analysis[J]. Wirel Commun Mob Comput.

[26] Baker S, Scharstein D, Lewis JP (2011). A database and evaluation methodology for optical flow[J]. Int J Comput Vision.

[27] Barron JL, Fleet DJ, Beauchemin SS (1994). System and experiment performance of optical flow techniques[J]. Int J Comput Vision.

[28] Wang XS (2009). Echocardiography [M].

